# Cardiac Arrest Center – Zertifizierung stärkt Patientenzufluss durch den Rettungsdienst

**DOI:** 10.1007/s00063-022-00939-z

**Published:** 2022-07-12

**Authors:** Nadine Rott, Sabine Wingen, Dirk Müller, Bernd W. Böttiger

**Affiliations:** 1 Klinik für Anästhesiologie und Operative Intensivmedizin, Medizinische Fakultät, Universitätsklinikum Köln, Kerpener Str. 62, 50937 Köln, Deutschland; 2 Deutscher Rat für Wiederbelebung, Prittwitzstraße 43, 89070 Ulm, Deutschland; 3 FOM University of Applied Sciences, Agrippinawerft 4, 50678 Köln, Deutschland; 4 Institut für Gesundheitsökonomie und Klinische Epidemiologie, Medizinische Fakultät, Universitätsklinikum Köln, Gleueler Str. 176–178, 50935 Köln, Deutschland

**Keywords:** Herz-Kreislauf-Stillstand, Kardiopulmonale Reanimation, Rettungsdienst, Einweisungsverhalten, Wiederbelebung, Cardiovascular arrest, Cardiopulmonary resuscitation, Emergency medical services, Referral behavior, Emergency physicians

## Abstract

**Hintergrund:**

Seit 2015 empfehlen die internationalen Reanimationsleitlinien die Implementierung von spezialisierten Krankenhäusern (sog. Cardiac Arrest Center, CAC) für die Versorgung von Patienten mit außerklinischem Herz-Kreislauf-Stillstand.

**Ziel:**

Ziel der Studie war es, den potenziellen Einfluss der Zertifizierung von Kliniken als CAC auf das Einweisungsverhalten von Notärzten und Rettungsfachpersonal bei prähospital reanimierten Patienten zu untersuchen.

**Methoden:**

Eine webbasierte anonyme Befragung mit 20 Items wurde vom 15.05. bis zum 15.06.2018 in Deutschland durchgeführt. Zielgruppen waren im Rettungsdienst tätige Notärzte sowie Rettungsfachpersonal.

**Wesentliche Ergebnisse:**

Von 437 Teilnehmern wurden die Ergebnisse von 378 Befragten (*n* = 292 Notärzte, *n* = 86 Rettungsfachpersonen) in die statistische Analyse eingeschlossen. 75,1 % (*n* = 284) gaben an, dass die CAC-Zertifizierung von Krankenhäusern bei ihrem künftigen Einweisungsverhalten von Patienten mit präklinischem Herz-Kreislauf-Stillstand eine Rolle spielen würde. 78,3 % (*n* = 296) erwarteten, dass die CAC-Zertifizierung zu einer Verbesserung der Patientenversorgung führen wird. 78,8 % (*n* = 298) befürworteten die Einführung der CAC-Zertifizierung. Die Befragten würden eine zusätzliche Transportzeit von 16,3 min (95 %-KI: 15,2–17,3) akzeptierten, um ein CAC zu erreichen.

**Fazit:**

Die Zertifizierung von Kliniken als CAC hat das Potenzial, die Entscheidung des Rettungsdienstpersonals bei der Zuweisung von Patienten mit einem präklinischen Herz-Kreislauf-Stillstand zu beeinflussen. Aufgrund der limitierten zusätzlich akzeptablen Transportzeit zur Erreichung eines CAC bedarf es eines bundesweiten, engen Netzes zertifizierter Kliniken.

**Zusatzmaterial online:**

Zusätzliche Informationen sind in der Onlineversion dieses Artikels (10.1007/s00063-022-00939-z) enthalten.

## Abstract (en)

**Background:**

Since 2015, the international resuscitation guidelines recommend the implementation of specialized hospitals (so-called cardiac arrest centers, CAC) for patients with out-of-hospital cardiac arrest (OHCA).

**Objectives:**

The aim was to investigate the potential influence of hospital certification as a CAC on the decision of emergency medical service staff (EMS, including out-of-hospital emergency physicians and paramedics) when transporting OHCA patients to a hospital.

**Materials and methods:**

A web-based, anonymous questionnaire with 20 items was performed from 15 May 2018 to 15 June 2018 in Germany. Target groups were out-of-hospital emergency physicians and paramedics.

**Results:**

Of 437 respondents, 378 responses (*n* = 292 emergency physicians, *n* = 86 paramedics) were included in the statistical analysis. In all, 75.1% (*n* = 284) indicated that CAC certification of hospitals would matter in their own transportation decisions for OHCA patients in future transportations, 78.3% (*n* = 296) expected that CAC certification will result in an improvement in patient care, and 78.8% (*n* = 298) were in favor of implementing the CAC certification. Respondents would accept an additional 16.3 min (95% confidence interval 15.2–17.3) of transportation time to reach a CAC.

**Conclusions:**

Certification of hospitals as CAC has the potential to influence emergency medical personnel decisions about which hospital to transport OHCA patients to. Due to the limited additional acceptable transport time to reach a CAC, a close network of certified hospitals is needed nationwide.

## Einführung

Seit 2015 empfehlen die internationalen Wiederbelebungsleitlinien die Implementierung von spezialisierten Krankenhäusern (sog. Cardiac Arrest Center/CAC) für Patienten nach einem außerklinischen Herz-Kreislauf-Stillstand. Ziel dieser Untersuchung war es, den Einfluss der Zertifizierung eines Krankenhauses als CAC auf die Entscheidung für das Zielkrankenhaus des Rettungsdienstpersonals beim Transport von prähospital reanimierten Patienten zu untersuchen.

## Hintergrund und Ziel

Im Jahr 2020 überlebten in Deutschland nur 10,5 % der initial reanimierten Patienten mit außerklinischem Herz-Kreislauf-Stillstand bis zur Entlassung aus dem Krankenhaus [8]. Eine europäische Studie ergab eine durchschnittliche Überlebensrate von 8 % für diese Patienten [9]. Die Überlebenskette weist auf die Bedeutung der prähospitalen Versorgung für das Überleben von prähospital reanimierten Patienten hin [16]. Die Qualität der Versorgung im Krankenhaus hat einen großen Einfluss auf das Überleben der Patienten und ist somit ein elementarer Schritt des Versorgungsprozesses und Teil dieser Kette. Seit 2015 empfehlen die internationalen Reanimationsleitlinien die Einführung von Spezialkliniken für prähospital reanimierte Patienten, um die Versorgungsqualität weiter zu verbessern und zu objektivieren, sog. Cardiac-Arrest-Zentren (CAC; [1, 10]). In den jüngsten Reanimationsleitlinien 2021 wurde die Bedeutung der Überlebenskette – und der CAC als ein Teil davon – durch die Einführung eines neuen Kapitels „Systeme, die Leben retten“ hervorgehoben [16].

Dieser Empfehlung folgend hat ein interdisziplinäres Team aus Anästhesiologen, Kardiologen und Intensivmedizinern unter der Schirmherrschaft des German Resuscitation Council (GRC) im Jahr 2017 die Basiskriterien für eine CAC-Zertifizierung in Deutschland festgelegt [14, 15].

Die Basiskriterien definieren Struktur‑, Prozess- und Ergebnisqualität für CAC-zertifizierte Krankenhäuser. Darüber hinaus sollen Behandlungspfade für prähospital reanimierte Patienten etabliert werden, wobei die Ergebnisse standardisiert erfasst und in der Rettungskette rückwärts kommuniziert werden müssen. Die Basiskriterien für CAC wurden von der Deutschen Gesellschaft für Anästhesiologie und Intensivmedizin (DGAI), der Deutschen Gesellschaft für Kardiologie – Herz- und Kreislaufforschung (DGK) und der Deutschen Gesellschaft für Internistische Intensivmedizin und Notfallmedizin (DGIIN) verabschiedet [14, 15].

Insgesamt zielt die Zertifizierung von Krankenhäusern als CAC darauf ab, die Qualität der Versorgung von prähospital reanimierten Patienten nach der Reanimation zu verbessern, da strukturelle Merkmale der Krankenhäuser das Ergebnis zu beeinflussen scheinen: Es hat sich gezeigt, dass die Größe eines Krankenhauses und die Anzahl der in einem Krankenhaus behandelten prähospital reanimierten Patienten für das Überleben eine prognostische Bedeutung haben [4, 5] und dass die Möglichkeit einer perkutanen Koronarintervention die Überlebenschancen erhöht [3].

Darüber hinaus soll die CAC-Zertifizierung den Rettungsdienst bei der Entscheidung für ein Zielkrankenhaus unterstützen.

Die vorliegende Studie untersucht erstmals den potenziellen Einfluss einer CAC-Zertifizierung auf das Einweisungsverhalten des Rettungsdienstpersonals.

## Methodik

### Studiendesign

Eine prospektive, anonyme Onlinebefragung wurde in einem festgelegten Zeitraum vom 15.05.2018 bis zum 15.06.2018 in Deutschland durchgeführt. Zu diesem Zeitpunkt waren die CAC-Zertifizierungskriterien bereits veröffentlicht [1], während der Auditierungsprozess für Krankenhäuser noch nicht begonnen hatte.

### Datenquelle/Datenerhebung

Der Fragebogen wurde mit SurveyMonkey (SurveyMonkey Inc., San Mateo, CA, USA), einer webbasierten Befragungssoftware, erstellt. Alle 11 Landesarbeitsgemeinschaften der Notärzte in Deutschland wurden gebeten, den Fragebogenlink per E‑Mail an ihre Mitglieder zu versenden. Darüber hinaus erhielten sie die Einladung, den webbasierten Fragebogen auch an ihre lokalen Rettungsdienstverbände weiterzuleiten.

### Teilnehmer

Die Zielgruppe der Studie war das Rettungsdienstpersonal (Notärzte und Rettungsfachpersonal) in Deutschland.

Notärzte sind die primären Entscheidungsträger bei der Versorgung von Patienten mit präklinischem Herz-Kreislauf-Stillstand (Herz-Kreislauf-Stillstand als „Notarztindikation“ [19]). Das Rettungsfachpersonal umfasst Rettungssanitäter, Rettungsassistenten und Notfallsanitäter. Befragt wurde sowohl Personal mit Anbindung an ein bestimmtes Krankenhaus als auch Personal ohne diese Anbindung.

### Fragebogen

Die Erstellung des Fragebogens basierte auf einer Literaturrecherche, aus der die folgenden 4 Fragebogenthemen abgeleitet wurden:

1. Erwartet das Rettungsdienstpersonal selbst, dass die Zertifizierung einen Einfluss auf ihr Einweisungsverhalten hat?
2. Welche Aspekte beeinflussen Ihre derzeitige Einweisungsentscheidung?
3. Erwarten Sie einen Nutzen der CAC-Zertifizierung für die Patientenversorgung?
4. Sind Entfernung und die wahrgenommene Qualität der Zielkliniken derzeit ausschlaggebend für die Einweisungsentscheidung?

Der Fragebogen wurde final durch 4 Experten (darunter ein Kardiologe, Notärzte und Mitglieder der GRC CAC-Arbeitsgruppe) kontrolliert. Es erfolgte ein Pretest durch 2 Personen aus der Zielgruppe.

Der Fragebogen bestand aus 20 Items (Supplementärmaterial). Den Befragten wurden 14 Items gestellt, die kontextbezogene Fragen zu den 4 oben genannten Aspekten und Annahmen enthielten. Außerdem wurden die Merkmale der Befragten i) Berufsgruppe des Arztes; ii) (Haupt‑)Einsatzgebiet; iii) Zugehörigkeit zu einer Klinik und prozentualer Anteil der Patienten, die in dieses Klinikum gebracht wurden; iv) Arbeitserfahrung (Notarzt seit) und v) medizinischer Fachbereich abgefragt.

Bei den meisten kontextbezogenen Fragen handelte es sich um geschlossene Ja‑/Nein‑/Weiß-nicht-Fragen. Zwei Fragen wurden in Form eines Schiebereglers gestellt, da sie sich auf Kompromisse bezogen. Zwei offene Fragen boten die Möglichkeit, Aspekte zu äußern, die in den vorherigen Fragen vernachlässigt wurden.

### Statistische Analyse

Die Ergebnisse werden auf einer aggregierten Ebene (Rettungsdienst) und für die jeweiligen Untergruppen (Ärzte und Rettungsfachpersonal) berichtet. Unvollständige Fragebögen (mindestens eine inhaltliche Frage unbeantwortet oder keine Zuordnung zu einer der beiden Zielgruppen möglich) wurden von der statistischen Auswertung ausgeschlossen.

Für die statistische Analyse wurde SPSS (IBM SPSS Statistics 25, IBM, USA) verwendet. Nominale Variablen wurden durch absolute und relative Zahlen beschrieben. Bei metrischen Variablen wurden Mittelwert, 95 %-Konfidenzintervall (95 %-KI), Median, Interquartilsbereich (IQR), Standardabweichung, Minimal- und Maximalwerte angegeben. Darüber hinaus wurden der Mann-Whitney-U-Test für unabhängige metrische Variablen und der χ^2^-Test für nominale Variablen angewandt, um eine Subgruppenanalyse durchzuführen (Ärzte im Vergleich zu Rettungsfachpersonal). Als statistische Signifikanz wurde ein *p*-Wert ≤ 0,05 angenommen.

## Ergebnisse

**Figure 1:**
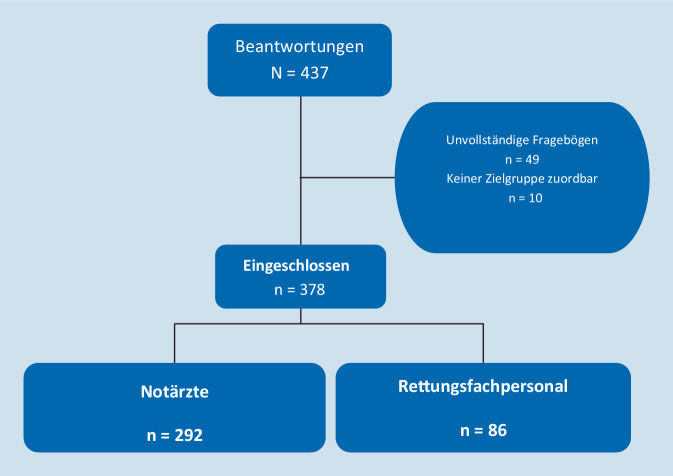

437 Teilnehmer nahmen an der Studie teil (siehe Abb. 1). 59 Antworten wurden ausgeschlossen, weil die Fragebögen unvollständig waren (*n* = 49) oder weil sie nicht einer der Zielgruppen zugeordnet werden konnten (*n* = 10). 378 Fragebögen wurden in die statistische Auswertung einbezogen (*n* = 292 Antworten von Notärzten; *n* = 86 von Rettungsfachpersonal).

Die Charakteristika der Befragten sind in Tab. 1 dargestellt.

**Table 1:** 

		Gesamt/Rettungsdienst		Notärzte		Rettungsfachpersonal		
		*N*	%	*N*	%	*N*	%	*p*-Wert^a^
Gesamt	–	378	100	292	77,3	86	22,8	–
Einsatzgebiet	Land	81	21,4	53	18,2	28	32,6	0,004
	Stadt	124	32,8	99	33,9	25	29,1	0,401
	Beides	170	45,0	137	46,9	33	38,4	0,161
	Sonstiges	3	0,8	3	1,0	0	0	0,345
Zugehörigkeit zu einer Klinik	Ja	192	50,8	155	53,1	37	43,0	0,101
	Nein	168	44,4	123	42,1	45	52,3	0,094
	Sonstiges	18	4,8	14	4,8	4	4,7	0,956
Arbeitserfahrung/Notarzt seit	≤ 1 Jahr	–	–	23	7,8	–	–	–
	> 1 Jahr	–	–	269	92,2	–	–	–
Medizinischer Fachbereich	Anästhesiologie	–	–	188	64,4	–	–	–
	Innere Medizin	–	–	32	11,0	–	–	–
	Kardiologie	–	–	10	3,4	–	–	–
	Sonstiges	–	–	62	21,3	–	–	–

^a^χ^2^-Test Notärzte und Rettungsfachpersonal

## Einfluss der CAC-Zertifizierung auf das Einweisungsverhalten

Insgesamt antworteten 75,1 % des Rettungsdienstpersonals, dass die CAC-Zertifizierung ihre eigene Entscheidung für ein Zielkrankenhaus in der Zukunft beeinflussen würde (73,6 % Notärzte vs. 80,2 % Rettungsfachpersonal; *p* = 0,213).

Das Rettungsfachpersonal war stärker überzeugt, dass alle prähospital reanimierten Patienten in ein CAC-zertifiziertes Krankenhaus gebracht werden sollten, als die teilnehmenden Notärzte (54,1 % vs. 75,6 %; *p* < 0,001). Andere Qualitätszertifizierungen, die in der Vergangenheit eingeführt wurden, hatten bereits bei 82,3 % des Rettungsdienstpersonals die Entscheidung für das Zielkrankenhaus beeinflusst, wobei es keine signifikanten Unterschiede zwischen den Gruppen gab (80,5 % vs. 88,4 %; *p* = 0,092).

Beide Gruppen stimmten darin überein, dass sie wissen, welche Krankenhäuser für die Behandlung von prähospital reanimierten Patienten am besten geeignet sind (89,9 %), und dass sie keine Schwierigkeiten haben, das für prähospital reanimierte Patienten geeignetste Krankenhaus zu ermitteln (79,1 %). Sie befürworteten aber dennoch die Einführung von CAC mit 78,8 % Zustimmung und würden dementsprechend mehr Patienten zu Krankenhäusern mit CAC-Zertifizierung transportieren (64,6 %). Das Rettungsfachpersonals befürwortete die Einführung von CAC sogar mit 91,9 % (*p* = 0,001) und würde mit 76,7 % (*p* = 0,007) mehr Patienten zu CAC transportieren.

### Akzeptierte zusätzliche Transportzeit

**Figure 2:**
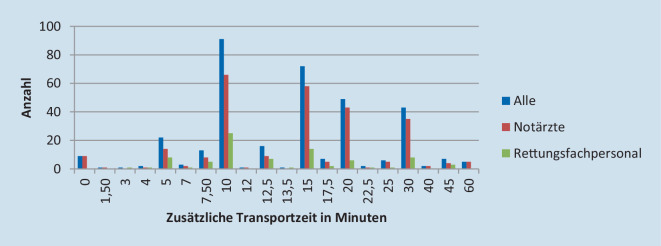

Es besteht ein signifikanter Unterschied zwischen den Gruppen hinsichtlich der akzeptierten zusätzlichen Transportzeit, um einen prähospital reanimierten Patienten zu einem CAC zu bringen: Insgesamt würden im Mittel 16,3 zusätzliche Minuten akzeptiert (95 %-KI 15,19–17,33); Notärzte würden 16,76 (95 %-KI 15,5–18,03) Minuten und Rettungsfachpersonal 14,64 (95 %-KI 12,67–16,62) Minuten zusätzlich akzeptieren (*p* = 0,028). Abb. 2 zeigt die Verteilung der Antworten der Befragten in Bezug auf die akzeptierte zusätzliche Transportzeit.

### Situation vor der Zertifizierung

Die Umfrage ergab, dass sowohl Notärzte als auch das Rettungsfachpersonal bei ihren Einweisungsentscheidungen auf die Qualität und die Einrichtungen/Ausstattung der Krankenhäuser achten (96,3 % Zustimmung). Sie halten dies für wichtiger als die Entfernung zum Krankenhaus. Notärzte legen signifikant mehr Wert auf Qualität und Ausstattung als das Rettungsfachpersonal (*p* = 0,038).

Sowohl Notärzte als auch das Rettungsfachpersonal hatten mit Kapazitätsproblemen zu kämpfen (d. h. sie können Patienten oft nicht in das Krankenhaus ihrer Wahl einweisen [51,2 % vs. 35,3 %, *p* = 0,008]).

### Sicht auf die Zertifizierung

Sowohl Notärzte als auch das Rettungsfachpersonal stimmten zu, dass die CAC-Zertifizierung ihrer Erwartung nach die Qualität der Versorgung von prähospital reanimierten Patienten insgesamt verbessern wird (78,3 % Zustimmung), wobei das Rettungsfachpersonal signifikant häufiger zustimmt (75,7 % vs. 87,2 %, *p* = 0,023). Die vermuteten Gründe hierfür unterscheiden sich zwischen den Gruppen und sind im Supplementärmaterial detailliert aufgezeigt.

## Diskussion

Die Ergebnisse der Befragung zeigen, dass die Mehrheit des Rettungsdienstpersonals einen Einfluss der CAC-Zertifizierung auf zukünftige Einweisungsentscheidungen erwartet, und dass frühere Zertifizierungen ihre Entscheidungen in der Vergangenheit beeinflusst haben. Sie erwarten, dass die CAC die Qualität der Versorgung von prähospital reanimierten Patienten insgesamt verbessern werden, und befürworten die Einführung der Zertifizierung.

Die erwartete Verbesserung der Versorgungsqualität deckt sich mit der Empfehlung für CAC in den jüngsten Reanimationsleitlinien, die auf einem Systematic Review mit 22 Studien beruhen, das ergab, dass Patienten, die in CAC versorgt wurden, eine bessere Überlebensrate bis zur Krankenhausentlassung mit günstigen neurologischen Ergebnissen hatten [16, 20].

Obwohl sie angeben, bereits zu wissen, welches Krankenhaus das geeignetste ist, befürworten Mitarbeiter des Rettungsdienstes die Zertifizierung. Einige der Befragten waren überzeugt, qualitativ hochwertige Krankenhäuser zu kennen, aber dass dies für neue Kollegen nicht der Fall sei und diese die Zertifizierung zu Beginn ihrer Karriere oder in einem neuen Arbeitsbereich bräuchten. Außerdem kann sich die Zertifizierung auf die Krankenhäuser selbst auswirken, indem diese die Kriterien erfüllen möchten und dadurch die Qualität der Patientenversorgung verbessern.

Die Tatsache, dass die Einführung anderer Zertifizierungen (Stroke Unit, Traumazentren) in der Vergangenheit zu einer Verbesserung der Versorgungsqualität geführt haben [2, 11], deutet darauf hin, dass sie das Einweisungsverhalten beeinflussen konnten.

Das Rettungsdienstpersonal würde im Durchschnitt 16 min zusätzliche Transportzeit für ein CAC akzeptieren. Frühere Studien in Kanada untersuchten, wie viel zusätzliche Transportzeit akzeptabel wäre, und kamen zu dem Schluss, dass zusätzliche 14 min den positiven Effekt eines CAC ausgleichen würden [7] und dass im Durchschnitt drei zusätzliche Minuten erforderlich sind, um ein CAC zu erreichen [6]. Andere Studien kamen sogar zu dem Schluss, dass die Wahl eines CAC unabhängig von der zusätzlichen Transportzeit nie nachteilig ist: In Ontario konnte kein Zusammenhang zwischen Transportzeit und Überleben festgestellt werden (OR 1,01; 95 % 95 %-KI 0,99–1,05; [19]), in San Diego kein Zusammenhang zwischen der Transportzeit und dem Überleben bis zur Krankenhausaufnahme oder der Transportzeit und dem Überleben bis zur Krankenhausentlassung mit einer mittleren Transportzeit von acht Minuten [6]. In Arizona gab es keinen Zusammenhang zwischen Transportzeit und Überleben von fast 1200 Patienten mit einer mittleren Transportzeit von 6,9 min (OR 1,2; 0,77–1,80; [18]). Keine dieser Studien analysierte jedoch die Folgen sehr langer zusätzlicher Transportzeiten. Deshalb ist es für die Zertifizierung in Deutschland wichtig zu analysieren, wie viele CACs benötigt werden, um geringe zusätzliche Transportzeiten zu haben.

Diese Veröffentlichung soll als Motivation für eine ähnliche Studie dienen, nachdem die CAC-Zertifizierung in Deutschland erfolgreich eingeführt wurde [12, 13], die Empfehlung in den jüngsten Reanimationsleitlinien [16] wiederholt wurde und ein europäisches Positionspapier zu diesem Thema vorliegt [17]. Es sollte nun untersucht werden, ob sich die Meinung des Rettungsdienstpersonals nach Einführung der CAC-Zertifizierung geändert hat und ob das tatsächliche Verhalten des Rettungsdienstpersonals den in dieser Studie geäußerten Erwartungen entspricht – auch in Bezug auf die zusätzlich akzeptierte Transportzeit.

## Resümee

Die CAC-Zertifizierung scheint das Potenzial zu haben, das Einweisungsverhalten des Rettungsdienstes zu beeinflussen. Notärzte und Rettungsfachpersonal sind sich einig, dass dies einen Einfluss auf ihre Transportentscheidungen hätte. Somit besteht Potenzial zur Verbesserung der Patientenergebnisse. Allerdings muss die zusätzliche Transportzeit in Betracht gezogen werden. Da die akzeptierte zusätzliche Zeit für den Transport begrenzt ist, wäre ein dichtes Netz von CACs im ganzen Land erforderlich.

## Fazit für die Praxis

- Die CAC Zertifizierung hat das Potenzial, Einweisungsentscheidungen zu beeinflussen
- Es braucht ein dichtes Netz von CACs im Land, da die akzeptierte zusätzliche Transportzeit begrenzt ist

## Supplementary Information




