# Supplementary material for: Cardiac arrest centers—certification fosters inflow of patients by emergency medical services
Source: Med Klin Intensivmed Notfmed. 2022 Jul 12;118(4):263–8. [Article in German] doi: 10.1007/s00063-022-00939-z (PMC10160140; doi:10.1007/s00063-022-00939-z)
Supplement: Supplementary file 1 [file 63_2022_939_MOESM1_ESM.docx]

**Supplementär-Material**

| Frage | | Antworten | Alle | | Notärzte | | Rettungsfachpersonal | | P-Wert* (ja) |
| --- | --- | --- | --- | --- | --- | --- | --- | --- | --- |
|  | |  | N=378 | % | N=292 | % | N=86 | % |  |
| Spielt für Sie Ihr Wissen zu Ausstattung und Versorgungsqualität der einzelnen Krankenhäuser bei der Entscheidung über Einlieferung in ein bestimmtes Krankenhaus eine Rolle? | | Ja | 364 | 96,3 | 281 | 96,2 | 83 | 96,5 | 0,904 |
|  |  | Nein | 9 | 2,4 | 9 | 3,1 | 0 | 0 |  |
|  |  | Weiß nicht | 5 | 1,3 | 2 | 0,7 | 3 | 3,5 |  |
| Kommt es regelmäßig vor, dass Sie aufgrund der Kapazitätsauslastung den prähospital reanimierten Patienten nicht in das erste Krankenhaus Ihrer Wahl bringen können? | | Ja | 147 | 38,9 | 103 | 35,3 | 44 | 51,2 | **0,008*** |
|  |  | Nein | 200 | 52,9 | 167 | 57,2 | 33 | 38,4 |  |
|  |  | Weiß nicht | 31 | 8,2 | 22 | 7,5 | 9 | 10,5 |  |
| Würde eine solche Zertifizierung zukünftig bei Ihrer Entscheidung für ein Zielkrankenhaus eine Rolle spielen? | | Ja | 284 | 75,1 | 215 | 73,6 | 69 | 80,2 | 0,213 |
|  |  | Nein | 59 | 15,6 | 49 | 16,8 | 10 | 11,6 |  |
|  |  | Weiß nicht | 35 | 9,3 | 28 | 9,6 | 7 | 8,1 |  |
| Wären Sie dafür, dass zukünftig alle prähospital reanimierten Patienten nur in ein CAC gebracht werden? | | Ja | 223 | 59,0 | 158 | 54,1 | 65 | 75,6 | **<0,001*** |
|  |  | Nein | 110 | 29,1 | 99 | 33,9 | 11 | 12,8 |  |
|  |  | Weiß nicht | 45 | 11,9 | 35 | 12,0 | 10 | 11,6 |  |
| Es gibt bereits Kliniken, die sich Cardiac Arrest Center oder ähnliches nennen, obwohl es noch keine einheitliche Definition dieses Begriffes gibt. Hat dies Ihre Entscheidung bereits beeinflusst? | | Ja | 80 | 21,2 | 61 | 20,9 | 19 | 22,1 | 0,810 |
|  |  | Nein | 99 | 26,2 | 82 | 28,1 | 17 | 19,8 |  |
|  |  | Weiß nicht/Kenne keine | 199 | 52,6 | 149 | 51,0 | 50 | 58,1 |  |
| Haben in der Vergangenheit die Einführung anderer Zertifizierungen (Traumazentrum, Stroke Unit…) Ihr Einweisungsverhalten beeinflusst? | | Ja | 311 | 82,3 | 235 | 80,5 | 76 | 88,4 | 0,092 |
|  |  | Nein | 58 | 15,3 | 49 | 16,8 | 9 | 10,5 |  |
|  |  | Weiß nicht | 9 | 2,4 | 8 | 2,7 | 1 | 1,2 |  |
| Erwarten Sie, dass CAC die Versorgungsqualität von Patienten insgesamt verbessern werden? | | Ja | 296 | 78,3 | 221 | 75,7 | 75 | 87,2 | **0,023*** |
|  |  | Nein | 46 | 12,2 | 42 | 14,4 | 4 | 4,7 |  |
|  |  | Weiß nicht | 36 | 9,5 | 29 | 9,9 | 7 | 8,1 |  |
| Erwarten Sie, dass sie die Versorgungsqualität verbessern, weil: | Es Forschung ankurbelt? | Ja | 204 | 54,0 | 142 | 48,6 | 62 | 72,1 | **<0,001*** |
|  |  | Nein | 139 | 36,8 | 120 | 41,1 | 19 | 22,1 |  |
|  |  | Weiß nicht | 35 | 9,3 | 30 | 10,3 | 5 | 5,8 |  |
|  | Mit einer höheren Fallzahl innerhalb einer Klinik die Qualität steigt? | Ja | 330 | 87,3 | 251 | 86,0 | 79 | 91,9 | 0,149 |
|  |  | Nein | 37 | 9,8 | 32 | 11,0 | 5 | 5,8 |  |
|  |  | Weiß nicht | 11 | 2,9 | 9 | 3,1 | 2 | 2,3 |  |
|  | Mehr Häuser versuchen die Kriterien zu erfüllen? | Ja | 195 | 51,6 | 157 | 53,8 | 38 | 44,2 | 0,118 |
|  |  | Nein | 139 | 36,8 | 105 | 36,0 | 34 | 39,5 |  |
|  |  | Weiß nicht | 44 | 11,6 | 30 | 10,3 | 14 | 16,3 |  |
|  | Es weniger Fehltransporte von Patienten gibt? | Ja | 203 | 53,7 | 158 | 54,1 | 45 | 52,3 | 0,771 |
|  |  | Nein | 140 | 37,0 | 110 | 37,7 | 30 | 34,9 |  |
|  |  | Weiß nicht | 35 | 9,3 | 24 | 8,2 | 11 | 12,8 |  |
| Stimmen Sie den folgenden Aussagen zu? | Mir fällt es schwer einzuschätzen, Eine Zertifizierung würde mir helfen. | Ja | 58 | 15,3 | 42 | 14,4 | 16 | 18,6 | 0,340 |
|  |  | Nein | 299 | 79,1 | 236 | 80,8 | 63 | 73,3 |  |
|  |  | Weiß nicht | 21 | 5,6 | 14 | 4,8 | 7 | 8,1 |  |
|  | Ich weiß auch ohne Zertifizierung welches Krankenhaus richtig ist. | Ja | 340 | 89,9 | 264 | 90,4 | 76 | 88,4 | 0,581 |
|  |  | Nein | 19 | 5,0 | 14 | 4,8 | 5 | 5,8 |  |
|  |  | Weiß nicht | 19 | 5,0 | 14 | 4,8 | 5 | 5,8 |  |
|  | Ich würde mehr Patienten in CAC zertifizierte Häuser bringen. | Ja | 244 | 64,6 | 178 | 61,0 | 66 | 76,7 | **0,007*** |
|  |  | Nein | 100 | 26,5 | 93 | 31,8 | 7 | 8,1 |  |
|  |  | Weiß nicht | 34 | 9,0 | 21 | 7,2 | 13 | 15,1 |  |
|  | Ich befürworte die Einführung einer Zertifizierung. | Ja | 298 | 78,8 | 219 | 75,0 | 79 | 91,9 | **0,001*** |
|  |  | Nein | 57 | 15,1 | 52 | 17,8 | 5 | 5,8 |  |
|  |  | Weiß nicht | 23 | 6,1 | 21 | 7,2 | 2 | 2,3 |  |

* χ²-Test: Notärzte/Rettungsfachpersonal

Antworten der Befragten auf aggregierten Level sowie differenziert nach den beiden Gruppen Notärzte und Rettungsfachpersonal sowie Test auf signifikante Unterschiede der Anteile der ja-Antworten zwischen diesen Gruppen

|  |  | Alle | Notärzte | Rettungsfachpersonal | P-Wert* |
| --- | --- | --- | --- | --- | --- |
| Was spielt bei Ihrer Entscheidung eine größere Rolle: Entfernung des Krankenhauses (0) oder Ausstattung, Qualität etc. (100)? | Mittelwert (95% CI) | 69,17 (67,40-70,93) | 70,3 (68,36-72,24) | 65,34 (61,28-69,40) | **0,038*** |
|  | Median (IQR) | 70 (61-79,25) | 70 (60-80) | 66 (52-77) |  |
|  | Std, Abweichung | 17,450 | 16,858 | 18,929 |  |
|  | Min/Max | 0/100 | 4/100 | 0/100 |  |
| Können Sie abschätzen wie viel % Ihrer Patienten Sie in Ihre zugehörige Klinik einweisen? | Mittelwert (95% CI) | 60,89 (57,76-64,01) | 60,03 (56,5-63,56) | 64,49 (57,58-71,39) | 0,200 |
|  | Median (IQR) | 65 (42-78) | 60,5 (40-75) | 70 (50-80) |  |
|  | Std, Abweichung | 22,593 | 22,867 | 21,307 |  |
|  | Min/Max | 0/100 | 10/100 | 0/90 |  |
| Was wird für Ihre Entscheidung in Zukunft wichtiger sein: Die Entfernung zum Krankenhaus (0) oder das zertifizierte Cardiac Arrest Center (100)? | Mittelwert (95% CI) | 63,47 (61,18-65,76) | 62,37 (59,72-65,01) | 67,22 (62,66-71,78) | 0,116 |
|  | Median (IQR) | 67 (50-78) | 66 (50-77) | 70,5 (54,25-80,5) |  |
|  | Std, Abweichung | 22,662 | 22,973 | 21,277 |  |
|  | Min/Max | 0/100 | 0/100 | 0/100 |  |
| Wie viel zusätzliche Transportzeit würden Sie in Kauf nehmen um einen prähospital reanimierten Patienten in ein CAC zu bringen (in Minuten)? | Mittelwert (95% CI) | 16,26 (15,19-17,33) | 16,76 (15,5 - 18,03) | 14,64 (12,67-16,62) | **0,028*** |
|  | Median (IQR) | 15 (10-20) | 15 (10-20) | 12,5 (10-12,5) |  |
|  | Std, Abweichung | 10,235 | 10,527 | 9,111 |  |
|  | Min/Max | 0/60 | 0/60 | 3/50 |  |

* Mann-Whitney-U-Test: Notärzte/Rettungsfachpersonal

Antworten der Befragten auf aggregierten Level sowie differenziert nach den beiden Gruppen Notärzte und Rettungsfachpersonal sowie Test auf signifikante Unterschiede der Anteile zwischen diesen Gruppen
